# Supplementary material for: Expert surgeons and deep learning models can predict the outcome of surgical hemorrhage from 1 min of video
Source: Sci Rep. 2022 May 17;12:8137. doi: 10.1038/s41598-022-11549-2 (PMC9114003; doi:10.1038/s41598-022-11549-2)
Supplement: Supplementary file 1 — Supplementary Legends. [file 41598_2022_11549_MOESM1_ESM.docx]

**Supplemental Figure 1.** Correlation (R^2^) between blood loss prediction from all 4 expert surgeon graders, model, and ground truth data.
