# Supplementary material for: Expert surgeons and deep learning models can predict the outcome of surgical hemorrhage from 1 min of video
Source: Sci Rep. 2022 May 17;12:8137. doi: 10.1038/s41598-022-11549-2 (PMC9114003; doi:10.1038/s41598-022-11549-2)

Supplemental Table 1

Skill Rating	Number of Ratings	Prediction Accuracy	Mean Absolute Error (Blood, mL)
1	13	0.92	461
2	16	0.56	195
3	18	0.61	236
4	19	0.63	297
5	14	0.79	131
*Significance**		0.21<br>0.04 (Pooled)	0.002
**Expert Confidence**			
1	0		
2.0	5	0.60	269
3.0	15	0.53	179
4.0	35	0.63	337
5.0	25	0.88	200
*Significance**		0.08<br>0.02 (Pooled)	0.07

Supplemental Table 1. Association between rated skill level (Likert scale, 1 = novice, 5 = master surgeon) and confidence (Likert scale, 1 = very uncertain, 5 = very confident) on accuracy of hemorrhage control prediction and blood loss estimates. *Significance testing: Chi-squared test was used for accuracy prediction (categorical), analysis of variance (ANOVA) used for continuous variable. Pooled calculations were conducted by aggregating 'moderate' ratings (2-4) and comparing to 'extreme' ratings (1 or 5). Model performance on 'moderate' trials: 80% (skill rating), 90% (confidence) (p=0.41, 0.14 respectively).

Cross-Stitch Trials	Ground Truth* (mL)	Blood Loss Prediction (mL)	Success Prediction
‘Best’ Version 1	75	462	Success
‘Best’ Version 2	75	417	Success
‘Best’ Version 3	75	328	Success
‘Best’ Version 4	75	364	Success
‘Best’ Version 5	75	386	Success
‘Best’ Version 6	75	473	Success
‘Worst’ Version 1	1262	793	Failure
‘Worst’ Version 2	1262	792	Failure
‘Worst’ Version 3	1262	794	Failure
‘Worst’ Version 4	1262	793	Failure
‘Worst’ Version 5	1262	792	Failure
‘Worst’ Version 6	1262	792	Failure
**Shifted-Input Trials**	**Critical Error**	**Time Frame (s)**	**Prediction**
Video 1	~65 seconds	0 – 60	Success
Video 1		50 – 110	Failure
Video 2	~65 seconds	0 – 60	Success
Video 2		50 - 110	Failure

Supplemental Table 2. Follow-up experiments conducted to investigate methodology of model. Cross-stitch trials had 20 second segments from the three best trials by blood loss stitched together to form a 1 min segment in various orders (‘Best’ Versions 1-6), and 20 seconds from the three worst trials by blood loss (‘Worst’ Versions 1-6). These would represent clear successes and clear failures and validates claims regarding what the model may be using to make predictions. Shifted input trials represent trials where surgeons and models were not shown the critical error, and all incorrectly assessed the surgeon. When given the critical error, the surgeons would clearly identify the trial as a failure, and we demonstrate similar performance in the model. *Ground truth data was derived from taking the average actual blood loss from the respective trials.

Appendix A
SOCAL Dataset Methodology:

Portions of the following sections are adapted from Donoho et al., 2021[1], Kugener et al., 2021[2]. Data is publicly available at the following link.

Experimental Setup:

Resident, fellow, and attending surgeons, including neurosurgery, and otorhinolaryngology–head and neck, were recruited for participation at nationwide educational courses between 2017 and 2020 (the North American Skull Base Society Annual Meeting, North American Skull Base Society Summer Skull Base Surgery Course, University of Southern California Annual Hands-On Comprehensive Neuro-Endoscopy Course, Emory Cranial Base Surgery Course, and Stryker Med-Ed Skull Cranial Surgery Course). The study was approved by the IRB of the University of Southern California.

A high-fidelity simulated operating room was constructed, including a surgical technician, surgical field, and simulated patient vital signs. A lightly embalmed human cadaveric head was prepared, and a standard endonasal endoscopic approach to the sella turcica was performed by study staff. Following cadaver perfusion at a standardized flow rate and physiological blood pressure using an artificial blood substitute, injury of the cavernous segment of the internal carotid artery (ICA) was induced by laceration. Participants were given standardized verbal instructions on the parameters, instruments, and goals of the simulation, but were not initially given specific technical instructions.

The protocol consisted of trial 1 (T1), followed by an educational intervention, and then trial 2 (T2) was performed. During T1 and T2, participants attempted to control the perfused ICAI using a variety of standard instruments and techniques (suction, cottonoid patties, and, ultimately, muscle patch control). Monitors showed simulated vital sign decompensation, and each trial ended when either hemostatic control was obtained using a muscle patch or simulated mortality occurred at 300 seconds (defined as 'trial failure'). Time to hemostasis (TTH, in seconds) and blood loss (BL, in mL) were evaluated for each trial. After T1, subjects received specific feedback from one of the course instructors (endoscopic endonasal approach experts) and watched a standardized video of a senior author (G.Z.) explaining the recommended stepwise technique of ICAI management (Video 1). T2 was then performed with feedback.

Data and Videos

Intraoperative video was taken from the Karl Storz Video Neuro-Endoscope used during each of these trials. A total of 143 videos from this nationwide educational intervention were recorded and saved. Videos were recorded at a frame rate of 30 frames per second (fps) and at a resolution of 1280x720 or 1920x1080. These videos are taken from multiple cadaveric heads, with different lighting, anatomy, laceration sites, camera resolutions, and brands of endoscopic instruments. The duration of the trials varies from 46 seconds to 5 minutes. These videos were down sampled from 30 frames-per-second (fps) to 1fps using ffmpeg, and were manually annotated to outline surgical instruments in each video frame using bounding boxes following previously published protocols using the open-sourced image annotation software VoTT.[3,4]

In conjunction with trial video recordings, "outcomes data" (e.g. blood loss, task success) and demographic data (e.g. training status, confidence) was recorded for each participant.

This collection of annotated videos and corresponding surgeon demographics and performance data is termed the Simulated Outcomes Following Carotid Artery Laceration (SOCAL) Video Dataset[2].

References

1. Donoho DA, Pangal DJ, Kugener G, et al. Improved surgeon performance following cadaveric simulation of internal carotid artery injury during endoscopic endonasal surgery: training outcomes of a nationwide prospective educational intervention. *Journal of Neurosurgery*. 2021;1(aop):1-9. doi:10.3171/2020.9.JNS202672

2. Kugener G, Pangal DJ, Zada G. Simulated Outcomes following Carotid Artery Laceration. Published online August 10, 2021. doi:10.6084/m9.figshare.15132468.v1

3. Pangal DJ, Kugener G, Shahrestani S, Attenello F, Zada G, Donoho DA. Technical Note: A Guide to Annotation of Neurosurgical Intraoperative Video for Machine Learning Analysis and Computer Vision. *World Neurosurg*. Published online March 12, 2021. doi:10.1016/j.wneu.2021.03.022

4. *Microsoft/VoTT*. Microsoft; 2020. Accessed July 7, 2020. https://github.com/microsoft/VoTT

Supplemental Figure 1:

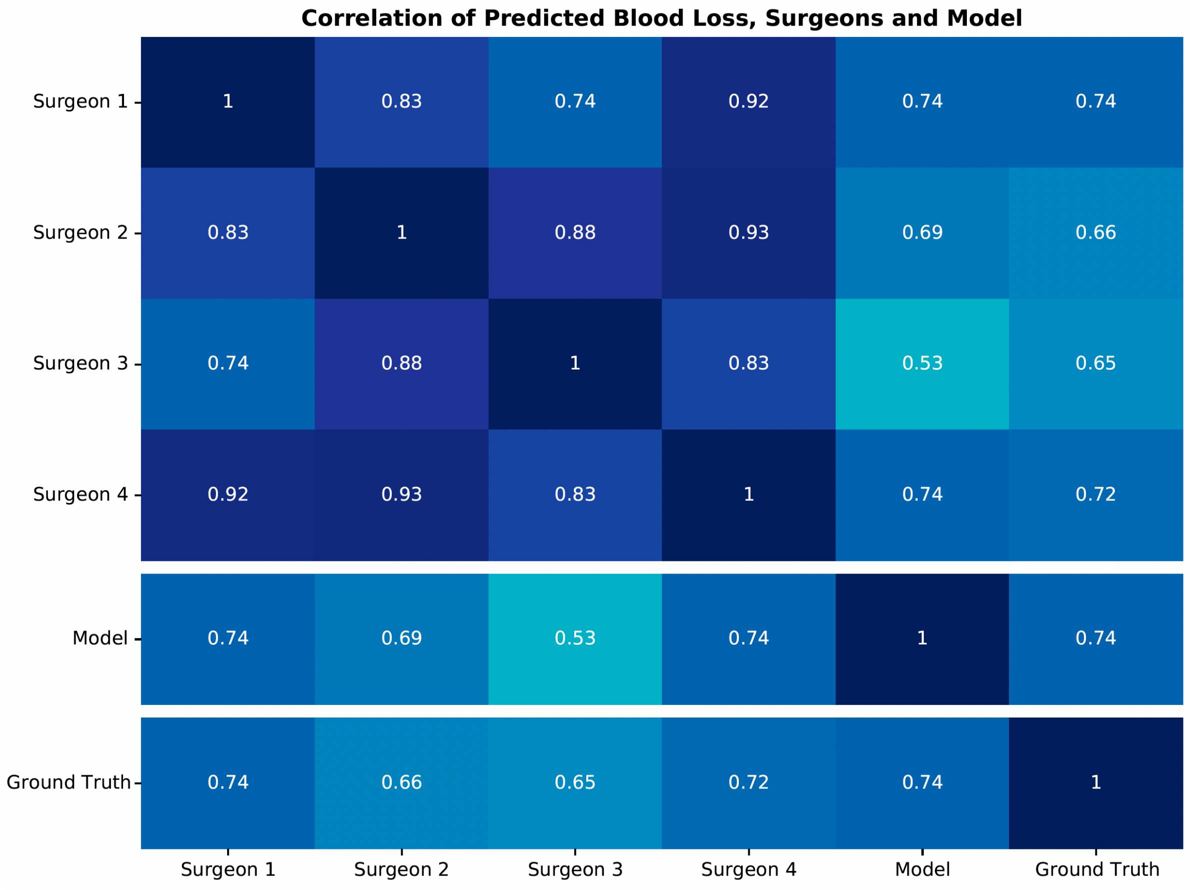

Supplement: Supplementary file 2 — Supplementary Information. [file 41598_2022_11549_MOESM2_ESM.pdf]
